# Factors associated with exclusive breastfeeding at hospital discharge: a study using data from the Georgian Birth Registry

**DOI:** 10.1186/s13006-020-00286-9

**Published:** 2020-05-13

**Authors:** Marie Sigstad Lande, Ingvild Hersoug Nedberg, Erik Eik Anda

**Affiliations:** Department of Community Medicine, UiT The Arctic University of Norway, Tromsø, Norway

**Keywords:** Exclusive breastfeeding, Patient discharge, Caesarean section, Neonatal intensive care units, Social determinants of health, Georgia (republic)

## Abstract

**Background:**

The World Health Organization recommends exclusive breastfeeding for six months, defined as no other solids or liquids besides breast milk and essential vitamins or medicines. Data about exclusive breastfeeding are limited in Georgia, and the information that exist are provided by national surveys, that present inconsistent numbers. Georgia has recently established a national birth registry, which includes information about early postpartum breastfeeding. The objective of this study was to identify factors associated with exclusive breastfeeding of term newborns at hospital discharge in Georgia, using national registry data.

**Methods:**

All live, singleton, term births registered in the Georgian Birth Registry in November and December 2017 were included, with a final study sample of 7134 newborns. Newborns exclusively breastfed at hospital discharge were compared with those who were not, and potential factors were assessed with logistic regression analysis. Hospital discharge normally occurred between 2 and 5 days postpartum.

**Results:**

The study identified several factors associated with nonexclusive breastfeeding of term newborns at hospital discharge in Georgia: maternal higher education compared to secondary education or less (Adjusted Odds Ratio [AOR] 0.75; 95% CI 0.59, 0.97), caesarean delivery compared to vaginal or assisted vaginal delivery (AOR 0.47; 95% CI 0.37, 0.60), birthweight < 2500 g compared to 3000–3499 g (AOR 0.51; 95% CI 0.27, 0.97), and admission to neonatal intensive care unit after delivery (AOR 0.02; 95% CI 0.02, 0.03). None of the following factors were associated with exclusive breastfeeding at discharge: mother’s age, marital status, Body Mass Index (BMI), parity, in vitro fertilization, maternal intrapartum complications and the sex of the newborn.

**Conclusions:**

To the authors’ knowledge, this is the first time determinants of exclusive breastfeeding at hospital discharge have been studied in Georgia. Several factors associated with nonexclusive breastfeeding at discharge were identified, most noteworthy were caesarean delivery and admission to neonatal intensive care unit. These findings are of importance to the Georgian health authorities and maternal/child non-governmental organizations.

## Background

The World Health Organization (WHO) recommends exclusive breastfeeding for the first six months of a child’s life, defined as no other solids or liquids besides breast milk and essential vitamins or medicines, and continued breastfeeding up to the age of two years or beyond [1]. To facilitate exclusive breastfeeding, the WHO promotes immediate skin-to-skin contact between mothers and newborns, and initiation of breastfeeding within one hour of delivery [2]. In low- and middle-income countries, only 37% of children under the age of six months are exclusively breastfed [3], despite the fact that breastfeeding is associated with numerous short- and long-term health advantages for both the mother and child. For mothers, long-term benefits include lower risk of breast cancer, ovarian cancer, and type II diabetes mellitus [4]. In children, breastfeeding has a protective effect against gastrointestinal and respiratory infections below five years of age [5], with a clear dose-response relationship [6] of long-term effects include lower odds of obesity and overweight in childhood and later life [7, 8].

Many factors affect breastfeeding; they can be maternal, newborn, or obstetric in nature and are often interlinked. Older age of the mother has been positively associated with exclusive breastfeeding (EBF) at discharge [9]. The relation between maternal age and EBF might be partly explained by parity, but this link is uncertain. Overweight and obesity has been associated with lower odds of exclusive breastfeeding [10], which may be related to both physical and psychosocial factors. Higher education and high socio-economic status of the mother are associated with increased rates of breastfeeding initiation and duration in high-income countries, for both exclusive and any breastfeeding [10–14], whereas in low- and middle-income countries this association is inversed [15–19]. Studies from all over the world indicate a negative association between caesarean delivery and breastfeeding [9, 14, 15, 20–25]. In addition to maternal and/or newborn distress, the effect of caesarean delivery on early breastfeeding might be related to delayed onset of lactation, problems with newborn suckling, disrupted early skin-to-skin contact and mother-newborn interaction, and postoperative hospital practices [23].

Located in the Caucasus region, Georgia is categorized as an upper-middle income developing country, ranked 70 of 188 countries in the 2017 Human Development Index [26]. Georgia has a population of 3.7 million; 87% consider themselves as ethnic Georgians and almost 11% are from the neighboring countries Armenia and Azerbaijan [27]. The infant mortality rate decreased from 22.5 in 2009 to 9.6 per 1000 livebirths in 2017, with a fertility rate of 2.1 in 2017 [27]. All medical facilities in Georgia are private. The beneficiaries of private insurance have decreased since Georgia introduced Universal Health Care in 2013. Basic obstetric care is free, which currently includes eight antenatal visits and the delivery. All Georgian mothers deliver at maternity wards with qualified healthcare personnel (99.9%) [28], and the average length of stay for vaginal delivery is 3–4 days and for caesarean delivery 5–6 days [29].

During the early 2000s, awareness increased about the advantages of breastfeeding in Georgia. In 2004, 14 out of 78 maternity wards in the country were designated as baby-friendly through the Baby-Friendly Hospital Initiative (BFHI), a global initiative to promote and support breastfeeding by WHO and the United Nations Children’s Fund [30]. However, today there are no baby-friendly hospitals left in Georgia due to lack of follow-up on the initiative and breastfeeding support after discharge is lacking at primary healthcare level. Data on breastfeeding practice in Georgia are limited. The information that does exist are provided by national surveys, which have presented inconsistent numbers. One survey from 2010 showed that 20% of mothers initiated breastfeeding within one hour of delivery [29], while another survey from the same year reported a proportion of 66% [31]. According to a survey from 2005, only 11% of children under six months were exclusively breastfed [32], whereas in 2010 this proportion was reported to be as much as 55% [31].

Georgia introduced a national birth registry in 2016, the Georgian Birth Registry (GBR) [33], which collects medical data from antenatal visits, as well as data from the delivery and postpartum period until discharge from the maternity ward/hospital. Reporting to the GBR is mandatory. Designated health professionals receive training in how to manage the register, and are then responsible for teaching the staff at their own facility. The GBR includes information on early postpartum breastfeeding. The objective of this study was to identify factors associated with exclusive breastfeeding of term newborns at hospital discharge in Georgia, using national registry data.

## Methods

**Fig. 1:**
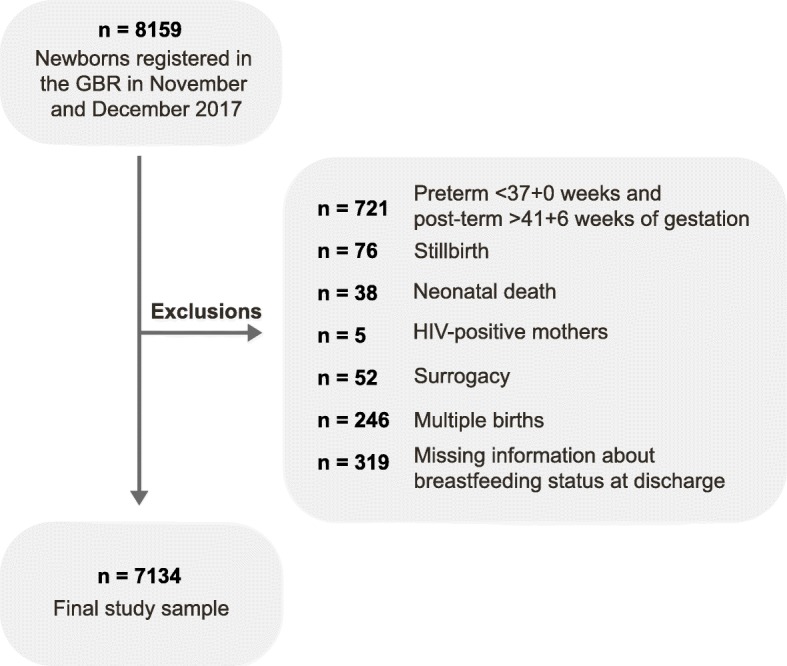
Flow chart of study sample inclusion and exclusion criteria

The study population consisted of all births registered in the GBR from November 1st to December 31st 2017 (*n* = 8159 newborns). Because this study looks at term newborns only (born between gestational age 37^+ 0^ and 41^+ 6^ weeks) [34], all preterm and post-term births were excluded (*n* = 721). Gestational age was estimated by the first day of the last menstrual period in 70.1% of the sample, with the rest being estimated by ultrasound. Stillbirths (*n* = 76) and neonatal deaths (*n* = 38) were also excluded, as were newborns with a HIV-positive mother (*n* = 5) or a surrogate mother (*n* = 52). Multiple births (*n* = 246) were excluded because these mothers face greater challenges to initiate and to continue breastfeeding [9, 35], as were newborns with missing information about breastfeeding status or unknown breastfeeding status at hospital discharge (*n* = 319). The final study sample consisted of 7134 live, singleton, term births, with information about breastfeeding at hospital discharge (Fig. 1).

### Variables

The outcome variable of interest was exclusive breastfeeding at hospital discharge, defined as the newborn receiving only breast milk the last feedings before discharge. Nonexclusive breastfeeding included documentation of mixed feeding, formula feeding, or parenteral feeding (intravenous administration of nutrition) in the last feedings before hospital discharge. Newborn discharge normally occurred between two to five days postpartum, with a median of three days for vaginal delivery and four days for caesarean delivery. Potential factors were selected based on the existing literature. The variables had to be available in the GBR dataset and reliable for the study purpose. Three categories of variables were considered: maternal, delivery, and newborn.

The maternal variables were mother’s age, marital status, education level, body mass index (BMI), parity and in vitro fertilization. Mother’s age at delivery was given in years and used in a continuous form in the regression analysis. For descriptive purposes, mother’s age was further categorized into five groups: < 20, 20–24, 25–29, 30–34, and ≥ 35 years. The youngest mother in the study sample was 14 years and the oldest was 52, which is biologically plausible; thus, no cut-offs were applied. Marital status was categorized as single, which included a small number of divorced women (*n* = 2), married, and unknown. Education level was categorized as completed secondary education or less, higher education, and unknown. BMI was computed by dividing the mother’s weight in kg at the first antenatal visit (before week 12 of pregnancy) by her height in m^2^. If the weight was not measured before week 12, the mother’s self-reported pre-pregnancy weight was used. BMI was then categorized in accordance with the WHO classification system [36]: underweight (< 18.5 kg/m^2^), normal weight (18.5- < 25 kg/m^2^), overweight (25- < 30 kg/m^2^) and obese (≥ 30 kg/m^2^). Extreme BMI values (< 15 and ≥ 60 kg/m^2^) were excluded from the statistical analysis [37]. Parity (i.e., the total number of previous live and stillbirths per woman), was categorized as 0, 1, 2, and ≥ 3 births. Three or more births were merged into one group because of low numbers. In vitro fertilization for the current birth was included as a dichotomous variable.

The delivery variables included were mode of delivery and maternal intrapartum complications. Mode of delivery was dichotomized as caesarean delivery (including both elective and emergency caesarean delivery) and vaginal delivery (including assisted delivery with forceps or vacuum, manual handling of breech delivery, and cases of episiotomy). Maternal intrapartum complications that may affect breastfeeding were constructed as *one* dichotomous variable and coded as “yes” if the mother experienced any of the following conditions: placenta praevia, placental abruption, meconium in amniotic fluid, umbilical cord prolapse, shoulder dystocia, uterine rupture, eclampsia during labor, retained placenta, uterine atony, or hemorrhage with total amount of bleeding > 500 ml.

Newborn variables were admission to neonatal intensive care unit (NICU) after delivery, birth weight and newborn sex. A NICU admission right after delivery leads to separation of mother and newborn, disrupting the early initiation of breastfeeding. Admission to NICU was included as a dichotomous variable. Unknown NICU admission was coded as missing due to the small numbers in the sample (*n* = 129). Birthweight was measured in grams and categorized as < 2500 g, 2500–2999 g, 3000–3499 g, 3500–3999 g, and ≥ 4000 g. Extreme values of < 500 g and > 7000 g were excluded from the analysis. The group that included the mean value (3000–3499 g) was set as the reference.

### Statistical analysis

The statistical analysis was conducted using R version 3.4.3 [38]. Demographic characteristics of the mothers and their newborns according to breastfeeding status at hospital discharge were compared using independent t-test, Wilcoxon rank-sum test, or chi-square test when appropriate. Logistic regression was used to assess potential factors associated with the newborn being exclusively breastfed at hospital discharge. The analyses were performed in accordance with the model building strategy described in Veierød et al. [39]. Variables that were significant at the 0.25 level in the univariable model were initially selected for inclusion in the multivariable model. Stepwise elimination was applied, and the full and reduced models were compared using the likelihood ratio test if more than one variable was removed. The changes in the coefficients (ß) of the remaining variables were computed to test if the removed variable(s) were needed to adjust for others in the model. If one coefficient changed by more than 20%, the variable was kept as a confounder in the final model. These steps were repeated until all variables left in the model were either significant or important confounders of other variables in the model. Only subjects without missing values on the variables in the final model were included (*n* = 6993). The following plausible interactions between the variables in the model were tested: parity and maternal age, parity and education level, parity and delivery mode, education level and delivery mode, maternal age and delivery mode, and NICU and birthweight. None of the interactions were significant. Case-wise diagnostics and multicollinearity were examined. The final model was tested for overall goodness-of-fit using the Hosmer-Lemeshow test. The results are presented with odds ratios (OR) and confidence intervals (CI), using the significance level 0.05.

## Results

Of the 7134 newborns in the study sample, 6583 (92.3%) were exclusively breastfed at hospital discharge and 551 were not. Maternal, delivery, and newborn characteristics differed by breastfeeding status at hospital discharge (Table 1).

**Table 1:** Maternal, delivery, and newborn characteristics according to breastfeeding status at hospital discharge^a^

	Exclusive breastfeeding at discharge	Nonexclusive breastfeeding at discharge	***p*** - value	Both groups combined	Total ***N***^**b**^
***N*** (mother-newborn pairs)	6583	551		7134	
**Mothers**					
Age at delivery in years mean (SD)	27.4 (5.63)	28.4 (5.75)	**< 0.001**	27.5 (5.65)	
Age at delivery in years %			**0.003**		
< 20	6.8	5.3		6.7	475
20–24	26.4	21.4		26.0	1855
25–29	33.2	33.9		33.3	2373
30–34	21.8	22.9		21.9	1560
≥ 35	11.8	16.5		12.2	871
Marital status %			0.13		
Single	13.1	11.3		13.0	924
Married	50.1	54.4		50.4	3598
Unknown	36.8	34.3		36.6	2612
Education level %			**< 0.001**		
Secondary school or less	56.7	48.5		56.0	3996
Higher education	34.9	45.6		35.8	2551
Unknown	8.4	6.0		8.2	586
BMI in kg/m^2^, median (25th–75th percentile)	22.9 (20.5–26.0)	23.2 (20.8–26.6)	0.14	22.9 (20.6–26.1)	
BMI in kg/m^2^, %			0.23		
< 18.5	7.5	7.0		7.5	465
18.5- < 25	60.3	59.5		60.3	3735
25- < 30	21.6	20.0		21.5	1334
≥ 30	10.5	13.4		10.7	663
Parity %			**0.03**		
0	39.8	43.0		40.1	2860
1	38.4	32.5		37.9	2707
2	16.9	18.0		17.0	1210
≥ 3	4.9	6.5		5.0	357
In vitro fertilization %	0.7	0.9	0.42	0.7	48
**Delivery**					
Mode of delivery %			**< 0.001**		
Vaginal delivery	58.3	46.2		57.3	4089
Caesarean delivery	41.7	53.8		42.7	3041
Maternal intrapartum complications %	4.8	5.4	0.58	4.9	347
**Newborns**					
Newborn sex %			0.88		
Female	48.3	48.7		48.3	3447
Male	51.7	51.3		51.7	3685
Birthweight in g, mean (SD)	3364 (426.5)	3274 (516.9)	**< 0.001**	3357 (434.8)	
Birthweight in g %			**< 0.001**		
< 2500	1.5	5.1		1.8	125
2500–2999	15.4	19.2		15.7	1121
3000–3499	44.0	41.9		43.9	3127
3500–3999	30.5	26.1		30.1	2148
≥ 4000	8.6	7.6		8.5	606
Admission to NICU %	1.8	43.7	**< 0.001**	4.4	308

^a^The Georgian Birth Registry, November–December 2017 (*n* = 7134)

*SD* standard deviation, *BMI* body mass index, *NICU* neonatal intensive care unit

^b^For some variables, the numbers do not add up to the total (*n* = 7134) because of missing values: education *n* = 7133, BMI *n* = 6197, mode of delivery *n* = 7130, newborn sex *n* = 7132, birthweight *n* = 7127, and admission to NICU *n* = 7005

In adjusted analyses, education level, mode of delivery, birthweight, and admission to NICU were identified as factors associated with nonexclusive breastfeeding at hospital discharge. Mothers with higher education were 25% less likely to exclusively breastfeed at hospital discharge compared to mothers with secondary education or less (AOR 0.75; 95% CI 0.59, 0.97). Newborns born by caesarean delivery were 53% less likely to be exclusively breastfed at hospital discharge compared to those born by vaginal delivery (AOR 0.47; 95% CI 0.37, 0.60). The newborns with the lowest birth weight (< 2500 g) had 49% lower odds of receiving exclusive breastfeeding at hospital discharge compared to those weighing 3000–3499 g (AOR 0.51; 95% CI 0.27, 0.97). In addition, admission to NICU after delivery was strongly associated with nonexclusive breastfeeding at hospital discharge. Newborns admitted to NICU were 98% less likely to be exclusively breastfed at hospital discharge compared to newborns who were not admitted (AOR 0.02; 95% CI 0.02, 0.03). None of the tested interaction terms were significant (Table 2).

**Table 2:** Odds ratios with 95% confidence intervals of exclusive breastfeeding at hospital discharge^a^

	Univariable analysis^b^	Multivariable analysis^**c**^
Mother’s age at delivery in years	**0.97 [0.96, 0.98]**	0.98 [0.96, 1.00]
Marital status		
Single	1.26 [0.96, 1.69]	–
Married	Reference	–
Unknown	1.17 [0.97, 1.41]	–
Education level		–
Secondary school or less	Reference	Reference
Higher education	**0.66 [0.55, 0.79]**	**0.75 [0.59, 0.97]**
Unknown	1.20 [0.84, 1.77]	1.28 [0.82, 2.09]
BMI in kg/m^2^		
< 18.5	1.06 [0.74, 1.56]	–
18.5- < 25	Reference	–
25- < 30	1.06 [0.84, 1.36]	–
≥ 30	0.77 [0.58, 1.03]	–
Parity		
0	Reference	Reference
1	**1.28 [1.04, 1.56]**	1.13 [0.86, 1.47]
2	1.01 [0.80, 1.30]	0.89 [0.64, 1.26]
≥ 3	0.81 [0.56, 1.18]	0.81 [0.49, 1.37]
In vitro fertilization for current birth	0.72 [0.31, 2.08]	–
Mode of delivery		
Vaginal delivery	Reference	Reference
Caesarean delivery	**0.61 [0.52, 0.73]**	**0.47 [0.37, 0.60]**
Maternal intrapartum complications	0.88 [0.61, 1.32]	–
Newborn sex		
Female	Reference	–
Male	1.02 [0.85, 1.21]	–
Birthweight in g		
< 2500	**0.28 [0.18, .44]**	**0.51 [0.27, 0.97]**
2500–2999	**0.76 [0.60, 0.97]**	0.83 [0.61, 1.14]
3000–3499	Reference	Reference
3500–3999	1.11 [0.90, 1.38]	1.17 [0.88, 1.55]
≥ 4000	1.07 [0.77, 1.53]	1.18 [0.78, 1.86]
Admission to NICU	**0.02 [0.02, 0.03]**	**0.02 [0.02, 0.03]**

^a^The Georgian Birth Registry, November–December 2017 (*n* = 7134)

*BMI* body mass index, *NICU* neonatal intensive care unit

^b^Complete case analysis: education level *n* = 7133, BMI *n* = 6197, mode of delivery *n* = 7130, newborn sex *n* = 7132, birthweight *n* = 7127, and admission to NICU *n* = 7005

^c^Complete case analysis: *n* = 6993

## Discussion

The study identified several factors associated with nonexclusive breastfeeding at hospital discharge: maternal higher education compared to secondary education or less, caesarean delivery compared to vaginal delivery, birth weight < 2500 g compared to 3000–3499 g, and admission to NICU.

In line with research from other low- and middle-income countries [15–19], the current study showed that mothers with higher education were less likely to exclusively breastfeed their newborns at hospital discharge. This finding is the opposite of what is seen in most high-income countries [10–14]. The association between education and exclusive breastfeeding in Georgia might be related to the work situation of mothers and the prospect of paid maternity leave. The unemployment rate among women in Georgia is low (11.2% in 2018, which is 2.7% lower than men) [40]. According to the Labour code of Georgia, a mother is entitled to 183 days (~ 26 weeks) of paid maternity leave financed by the state with a maximum of 1000 Georgian Lari [41], or around 305 Euro for the whole maternity leave. The legislation also states that employers and employees can agree on additional benefits [41]. However, for higher educated mothers with a well-paid job and for those without extra benefits, the compensation may be insufficient. If the mother returns to work early for economical or other reasons, exclusive breastfeeding in the postpartum period would probably not be a top priority.

Newborns born by caesarean delivery were less likely to be exclusively breastfed at hospital discharge compared to newborns born by vaginal or assisted vaginal delivery. This finding is in accordance with other studies [9, 21–23]. The proportion of caesarean deliveries is noticeably higher in Georgia (43.5% in 2016) [33] compared to the mean in European countries (25.2% in 2010) [42]. Considering this high rate, the negative association with early postpartum exclusive breastfeeding is of particular interest. Indeed, excessive rates of caesarean delivery is associated with several short- and long-term health risks for both the mother and newborn [43], and lower odds of exclusive breastfeeding at hospital discharge adds to the list of health risks. One of the *Ten steps to Successful Breastfeeding* in the BFHI is immediate skin-to-skin contact and early initiation of breastfeeding [44]. Without the designation of baby-friendly hospitals, Georgian hospitals and maternity wards may not pay enough attention to the early initiation of breastfeeding after a caesarean delivery. One systematic review suggested that adequate breastfeeding support after a caesarean delivery reduces the negative association between caesarean delivery and early initiation of breastfeeding entirely [45]. Another review found that, among mothers that successfully initiated breastfeeding, there was no difference in exclusive breastfeeding between babies delivered by caesarean and those born by vaginal delivery at six months [23]. These findings indicate that a supportive breastfeeding environment after a caesarean delivery, with proper postoperative pain management, could substantially improve the rates of exclusive breastfeeding at hospital discharge and later in the postpartum period. The Georgian Ministry of Health is addressing the high caesarean rates, and hospitals are under scrutiny to reduce their rates. However, it is too soon to evaluate the results of this intervention.

Newborn admission to a NICU had a large negative impact on exclusive breastfeeding at hospital discharge, a finding that is in line with other studies [46, 47]. This is an expected finding, as exclusive breastfeeding often cannot be prioritized in an intensive care setting. However, although the present study excluded premature newborns, the association between NICU admission and exclusive breastfeeding at hospital discharge was very strong (AOR 0.02; 95% CI 0.02, 0.03). Additionally, newborns with a birthweight < 2500 g were less likely to be exclusively breastfed at hospital discharge compared to newborns weighing 3000–3499 g. Low birthweight has been associated with lower odds of exclusive breastfeeding in previous studies as well [48]. An expansion of the BFHI has been developed for use in NICUs, where the *Ten Steps to Successful Breastfeeding* are adapted for preterm and sick newborns. The steps include early and prolonged mother-newborn skin-to-skin contact, also known as Kangaroo Mother Care, and support of early breastfeeding, with newborn physiological stability as the only criterion,- not newborn age, weight, or other criteria [49]. This is important, as early initiation of breastfeeding reduces the risk of neonatal mortality, also among low-birthweight newborns [50]. The BFHI expansion to NICUs recommends rooming-in, where mothers and newborns stay together in the NICU, as a step to facilitate continuous breastfeeding [49]. Rooming-in at NICUs may not be feasible in all settings, but the mother should then get the opportunity to stay close to the NICU.

The absolute majority of the term newborns in the study were exclusively breastfed at discharge (92.3%). The high proportion raises concern of reporting bias. Nurses and midwives should record newborn feeding twice a day, but we cannot be sure that all healthcare personnel follow the same procedures, with the risk of misclassification by type of newborn feeding at discharge. However, before applying the exclusion criteria, 85.0% of all newborns were exclusively breastfed at hospital discharge. Prevalence figures of exclusive breastfeeding at hospital discharge in other countries vary substantially, from 61.6% in Canada (only term newborns) [9], 82.7% in rural Western Australia [47], 86.9 -93.1% in the Czech Republic [51], to 93.5% in rural China (only healthy singletons) [52]. The prevalence figures from other countries support the plausibility of the high proportion of exclusive breastfeeding we observed in Georgia. Although it is previously reported a lower prevalence of breastfeeding in Georgia [29, 31, 32], the data are 10–15 years old and based on surveys. Furthermore, the data are not directly comparable as the surveys display the initiation and duration of exclusive breastfeeding. The rate of exclusive breastfeeding is likely much lower at 4–6 months, because the majority of mothers have returned to work or studies. A pilot follow-up program (up to 6 months) in the GBR was launched in 2019 in the region of Ajara in West Georgia. When this program is expanded, we will be able to conduct a follow-up study on the duration and potential obstacles of breastfeeding after hospital discharge.

The GBR was launched in 2016, and it takes time to establish good practices for proper reporting [33]. This is evident for some variables, for instance maternal intrapartum complications. Even though the variable merged several complications the mother may experience during delivery, the prevalence of these complications was low (total 4.9%), indicating a considerable underreporting of complications in the GBR. This underreporting can mask a potential effect of maternal intrapartum complications on exclusive breastfeeding at hospital discharge. Additionally, data are entered into the GBR by maternity wards all over the country, indicating that the healthcare personnel have different training in how to assess certain maternal and newborn conditions. The variable maternal intrapartum complications are particularly exposed, because many of the complications included in the variable are based on the judgement of the healthcare personnel attending the birth. Registry data is also vulnerable to data entry errors. Both of these problems could potentially introduce bias, but we do not know to what extent. Data on the validity of the variables in the GBR are still not available, hence the findings should be interpreted with some caution.

### Strengths and limitations

The GBR collects information on the absolute majority of births in Georgia, and participation in the registry is mandatory by law. The study population was limited to the two last months of 2017 because of structural changes in GBR variables, which were completed in late October of 2017. Selected variables (mother’s age, education level, BMI and mode of delivery) were compared between the study population before exclusions and total births in 2017. Based on these comparisons, there are no significant differences (< 1.5%) between the study sample and total births in 2017, thus the study sample is representative. Data from 2018 were not available at the time of submission.

Even though the study comprised a relatively large sample size (*n* = 7134), the number of newborns who were not exclusively breastfed at hospital discharge was small (*n* = 551) compared to those who were (*n* = 6583). This may have led to larger CIs than if the groups had been more equal in size or if the sample size had been bigger. The study adjusted for several confounders, but not for gestational age. Even though the study population only consisted of newborns at term, important differences between gestational age of 37^+ 0^ weeks and 41^+ 6^ weeks could still exist. In particular, gestational age is closely interlinked with the newborn factors birthweight and admission to NICU, and including gestational age as a confounder would likely have rendered these estimates more conservative than the presented results.

Several studies suggest a difference in breastfeeding outcomes between emergency and elective caesarean delivery [9, 23, 53]. However, these subgroups were not included in the study because of possible misclassification in favor of emergency deliveries in the GBR. In the initial population, 30.8% of all births were emergency caesarean deliveries and 14.3% were elective caesarean deliveries (data not shown). In comparison, the median proportion of emergency caesarean deliveries was 12.9% and for elective caesarean deliveries 10.7% in an aggregated study of European countries [42]. Had reliable data been available, separating the caesarean deliveries into elective and emergency would be important, as it would allow the adjustment for related variables like in vitro fertilization, maternal intrapartum complications, and birthweight.

Of the 8159 newborns in the initial study population, 135 were excluded due to missing or unknown breastfeeding status at hospital discharge. Compared to the study sample (*n* = 7134), these excluded newborns experienced a higher proportion of caesarean delivery and admission to NICU, both of which were factors associated with nonexclusive breastfeeding at hospital discharge. This suggests that there was a higher proportion of newborns who were not exclusively breastfed at discharge in the excluded cases. Hence, missing information on the main outcome variable could have led to an underestimation of the ORs for caesarean delivery and admission to NICU. Some of the included predictor variables had missing data; however, all variables had less than 0.1% missing, except for BMI with 13.1% missing. The cases with missing maternal BMI were compared with the included cases, and they did not differ significantly. Thus, the assumption is that the exclusion of these cases did not bias the effect estimates.

## Conclusions

To the authors’ knowledge, this is the first time exclusive breastfeeding at hospital discharge have been studied in Georgia, and certainly for the first time using national birth registry data. The study identified several factors associated with nonexclusive breastfeeding at hospital discharge in Georgia in term newborns: maternal higher education, caesarean delivery, low birthweight and admission to NICU after delivery. Potential steps to increase rates of exclusive breastfeeding are a re-introduction of the Baby-Friendly Hospital Initiative, reducing the high caesarean delivery rates in Georgia, as well as strengthening the length and economical support of the maternity leave. These findings will be valuable for national health authorities when setting new priorities in maternal and child health, as well as for non-governmental organizations working with breastfeeding dyads. Hopefully, the findings will increase awareness about breastfeeding in maternity wards and hospitals all over Georgia. The results could have validity in countries with high rates of caesarean delivery and similar health system structures as in Georgia.

## Data Availability

The National Centre for Disease Control and Public Health (NCDC), Tbilisi, provides data storage, administration, and quality control of the GBR. The datasets generated and analyzed during the current study are not publicly available without a written application and acceptance by NCDC.

## Abbreviations

BFHI: Baby-Friendly Hospital Initiative
GBR: Georgian Birth Registry
HIV: Human immunodeficiency virus
NICU: Neonatal Intensive Care Unit
WHO: World Health Organization
